# Diagnostic method for malignant pleural effusion distinguishing malignant mesothelioma from lung cancer using pleural carcinoembryonic antigen and hyaluronic acid levels

**DOI:** 10.1097/MD.0000000000028517

**Published:** 2022-01-07

**Authors:** Takeshi Saraya, Kosuke Ohkuma, Masachika Fujiwara, Haruyuki Ishii

**Affiliations:** a Kyorin University School of Medicine, Department of Respiratory Medicine, Japan; b Kyorin University School of Medicine, Department of Pathology, Japan.

**Keywords:** carcinoembryonic antigen, hyaluronic acid, lung cancer, malignant mesothelioma, pleural effusion

## Abstract

Malignant mesothelioma (MM) is difficult to diagnose because of the lack of parenchymal opacities, often revealing minimal or absent pleural thickening. Furthermore, pleural effusion has diverse differential diagnoses, including malignancies, infections, as well as collagen vascular and other benign diseases. In general practice, lung cancer (LC) is the most common malignancy causing pleural effusion; therefore, a simple method using pleural diagnostic markers to differentiate between LC and mesothelioma is crucial.

We retrospectively reviewed the data of 530 adult patients diagnosed with pleural effusion between January 2010 and December 2020 in an outpatient or inpatient setting. Patients with pathologically diagnosed MM or LC with cytologically positive (class IV or V) pleural effusion were analyzed, and the characteristics of these 2 diseases were compared.

During the study period, 27 patients diagnosed with MM and 100 patients diagnosed with LC were enrolled. Receiver operating characteristic curve analysis demonstrated that pleural carcinoembryonic antigen (CEA) and hyaluronic acid (HA) could discriminate MM from LC with an area under the curve of 0.925 (95% confidence interval [CI]: 0.879–0.972, *P* < .001) and 0.815 (95% CI: 0.686–0.943, *P* < .001), respectively. To diagnose MM, the accuracy of pleural HA >30,000 ng/mL revealed a sensitivity of 75.0%, specificity of 72.6%, and odds ratio of 7.94 (95% CI: 2.5–25.2, *P* = .001); pleural CEA <6.0 ng/mL revealed a sensitivity of 95.2%, specificity of 84.9%, smaller negative likelihood ratio of 0.06, and odds ratio of 112.5% (95% CI: 14.4–878.1, *P* < .001). Multiple logistic regression analysis revealed that these 2 parameters could discriminate MM from LC, with a hazard ratio of 23.6 (95% CI: 2.437–228.1, *P* = .006) and 252.3 (95% Cl: 16.4–3888.1, *P* < .001), respectively, and their combination had a high specificity of 98.3%.

Pleural CEA (≥6.0 ng/mL) can rule out MM with a high degree of certainty, and the positive results for combination of pleural CEA <6.0 ng/mL and HA >30,000 ng/mL can confirm MM with high specificity, prior to cytological or pathological examinations.

## 1 Introduction

Pleural effusion has differential diagnoses, which includes malignant pleural effusion (i.e., lung cancer [LC], malignant mesothelioma [MM], and pleural metastasis from other organs), tuberculous pleuritis or empyema/parapneumonic effusion caused by other bacteria, and benign transudative effusion because of hypoalbuminemia and heart or renal failure. We recently reported that the ratio of pleural lactate dehydrogenase to adenosine deaminase along with carcinoembryonic antigen (CEA) levels may be a valuable diagnostic marker for discriminating possible etiologies.^[1,2]^ The accuracy of cytological examination to diagnose malignant pleural effusion ranges from 40% to 87%.^[3–5]^ Furthermore, MM is one of the most challenging malignancies to diagnose. Therefore, this study aimed to identify pleural diagnostic markers to discriminate between LC and MM.

## 2 Methods

We retrospectively reviewed the data of 530 adult patients diagnosed with pleural effusion between January 2010 and December 2020 in an outpatient or inpatient setting. These patients either displayed signs of pleural fluid at the time of their first visit to the hospital (an 1100-bed tertiary care center in Tokyo) or developed it during hospitalization. Among them, we selectively enrolled patients diagnosed with MM and LC having cytologically positive pleural effusion (class IV or V) and all patients with LC were pathologically definite cases. This study was approved by the ethics board of Kyorin University (approval number: 685).

### 2.1 Statistical analysis

Numerical data were evaluated for normal distribution and equal variance using the Kolmogorov–Smirnov test and Levene median test, respectively. Categorical data are presented as percentages of the total or whole numbers, as appropriate. Statistical comparisons of non-parametric data were performed using the Mann–Whitney test, while categorical data were compared using Pearson chi-square test. All tests were two-sided, and statistical significance was set at *P* < .05. The cut-off point for the markers in the pleural fluid was determined as the minimum value of [(1-sensitivity)^2^ + (1-specificity)^2^]. Data were analyzed using Statistical Package for Social Science software (version 25.0; IBM Co., Japan) for Windows.

## 3 Results

### 3.1 Clinical characteristics of patients with MM and LC

During the study period, we enrolled 200 patients diagnosed with malignant pleural effusion, including patients with pathologically diagnosed MM (n = 27) and LC (n = 173) (Table 1). Although the male-to-female ratio was significantly higher in the MM group than in the LC group (*P* = .001), the median age was comparable. The MM group consisted of epithelial (n = 13), biphasic (n = 2), desmoplastic (n = 3), and unclassified (n = 9) pathological types, while the LC group consisted of adenocarcinoma (n = 152), squamous cell carcinoma (n = 7), small cell carcinoma (n = 10), and others (n = 4).

**Table 1:** Background.

	Mesothelioma (n = 27)	Lung cancer (n = 173)	*P* value
Gender (M/F)	25/2	106/67	.001^∗∗^
Age (yrs)	67 (59–78)	71 (65–79)	.346
Type			
	Epithelial (n = 13)	Adeno (ln = 152)	
	Biphasic (n = 2)	Squamous (n = 7)	
	Desmoplastic (n = 3)	Small cell carcinoma (n = 10)	
	Unclassified (n = 9)	Others (n = 4)	
Pleural effusion			
TCC (/μL)	925 (550–1300)	1250 (1038–1413)	.139
Lymphocyte (%)	27.5 (15.0–40.0)	59.0 (58.5–60)	.009^∗∗^
Neutrophil (%)	3.0 (1.0–5.0)	5.0 (3.5–5.0)	.748
Eosinophil (%)	1.0 (0–2.0)	0 (0–0.5)	.677
pH	8.2 (8.0–8.4)	8.0 (7.8–8.0)	.855
TP (g/dL)	4.4 (4.2–4.5)	4.8 (4.8–5.0)	.248
ALB (g/dL)	2.8 (2.8–2.9)	2.9 (2.7–3.0)	1
Glucose (mg/dL)	96 (89–103)	128 (80.5–185)	.099
LDH (U/L)	385 (358–412)	612 (566–753)	.836
ADA (U/L)	18.2 (16.9–19.5)	20.6 (20.6–22.6)	.003^∗∗^
LDH to ADA ratio	16.1 (10.2–33.2)	22.3 (15.2–35.9)	.078
T-Cholesterol (mg/dL)	67.0 (65.0–69.0)	88.0 (84.5–102)	.601
CEA (ng/mL)	1.4 (1.1–1.6)	196 (102–198)	<.001^∗∗∗^
CYFRA 21—1 (ng/mL)	110 (19.0–200)	150 (75.8–235)	.083
Hyaluronic acid (ng/mL)	111,900 (30,800–193,000)	8850 (8355–13,325)	<.001^∗∗∗^
Serum			
WBC (/μL)	6950 (6350–8150)	7400 (6100–9550)	.305
CRP (mg/dL)	3.6 (0.4–5.8)	1.4 (0.5–4.4)	.363
LDH (U/L)	196 (179–208)	231 (190–306)	.002
TP (g/dL)	7.1 (6.6–7.3)	6.9 (6.2–7.1)	.213
ALB (g/dL)	3.6 (3.1–3.9)	3.3 (2.9–3.7)	.256
T-Cholesterol (mg/dL)	169 (151–201)	164 (136–175)	.572
Glucose (mg/dL)	107 (98–129)	112 (96–137)	.561
CYFRA 21—1 (ng/mL)	1.6 (0.9–2.5)	4.1 (2.7–14.0)	.001^∗∗^

Serum data were comparable between the MM and LC groups, except for the known tumor markers for non-small cell lung carcinoma including lactate dehydrogenase (median: 196, interquartile range [IQR] 179–208 vs median: 231, IQR 190–306, *P* = .002) and CYFRA 21-1 (median: 1.6, IQR 0.9–2.5 vs median: 4.1, IQR 2.7–14.0, *P* = .001), which were significantly higher in the LC group than in the MM group (Table 1).

The ratios of lymphocyte to total cell counts, adenosine deaminase, and CEA levels in the LC group were significantly higher than those in the MM group—median: 59%, IQR 58.5–60.0 vs median 27.5%, IQR 15.04.0%, *P* = .009, median: 20.6, IQR 20.6–22.6 vs median 18.2, IQR 16.9–19.5, *P* = .003, median: 196, IQR 102–198 vs median 1.4, IQR 1.1–1.6, *P* < .001, respectively. The level of hyaluronic acid (HA) was significantly higher in the MM group (median: 111,990, IQR 30,800–193,000) than in the LC group (median: 8850, IQR 8355–13,325, *P* < .001) (Table 1).

### 3.2 Receiver operating curve for discrimination between MM and LC using pleural CEA and HA

**Figure 1:**
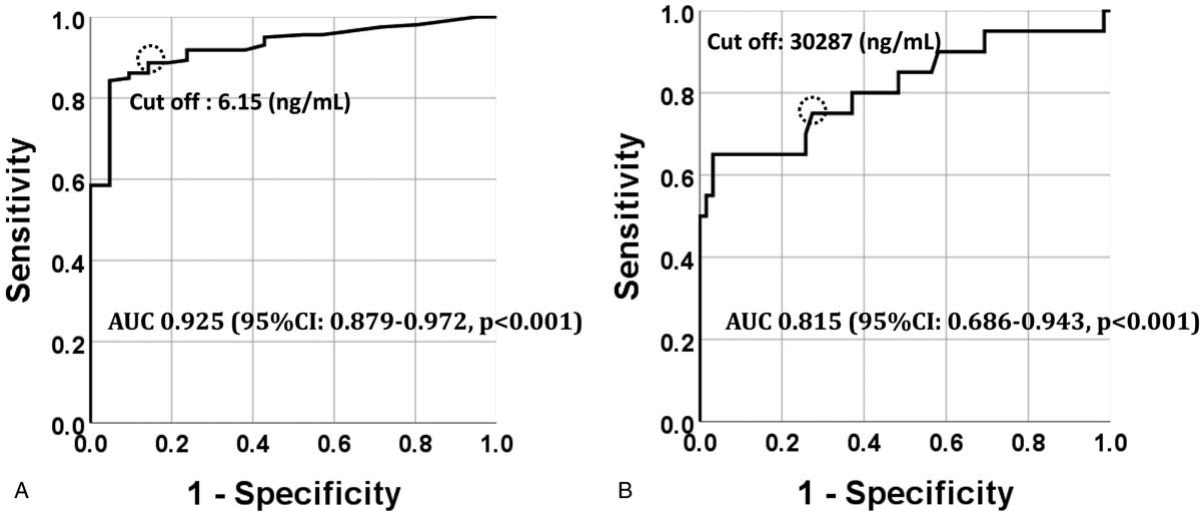
Pleural CEA (A) can discriminate malignant mesothelioma from lung cancer with an AUC of 0.925 (95% CI: 0.879–0.972, *P* < .001). The dotted circle represents a cut-off point of pleural CEA (6.15 ng/mL). Pleural HA (B) can discriminate mesothelioma from lung cancer with an AUC of 0.815 (95% CI: 0.686–0.943, *P* < .001). The dotted circle represents a cut-off point of pleural HA (30,2887 ng/mL). AUC = area under the curve, CEA = carcinoembryonic antigen, CI = confidence interval, HA = hyaluronic acid.

Receiver operating characteristic curve analysis demonstrated that pleural CEA and HA could discriminate MM from LC with an area under the curve of 0.925 (95% confidence interval [CI]: 0.879–0.972, *P* < .001) and 0.815 (95% CI: 0.686–0.943, *P* < .001), respectively. The optimal thresholds for CEA and HA were 6.15 (ng/mL) and 30,287 (ng/mL), respectively, with sensitivity and specificity of 84.3% and 95.2%, respectively (Fig. 1A, dotted circle), and 75.0% and 72.6%, respectively (Fig. 1B, dotted circle).

### 3.3 Diagnostic accuracy of combination of pleural CEA and HA for discrimination of MM from LC

We evaluated the diagnostic accuracy for differentiating MM from LC using the following parameters: pleural CEA <6.0 ng/mL, pleural HA >30,000 ng/mL, pleural CEA <6.0 ng/mL, and HA >30,000 ng/mL (Table 2). Using the value of pleural CEA, we could differentiate MM from LC with a sensitivity of 95.2%, specificity of 84.9%, and odds ratio of 112.5% (95% CI: 14.4–878.1, *P* < .001).

**Table 2:** Diagnostic accuracy of combination of pleural CEA and Hyaluronic acid for discrimination of malignant mesothelioma from lung cancer.

	Sensitivity	Specificity	PPV	NPV	PLR	NLR	Odds ratio (95% CI)	AUC	*P* value
CEA < 6.0 (ng/mL)	95.2	84.9	45.5	99.2	6.3	0.06	112.5 (14.4–878.1)	0.901	<.001
Hyaluronic acid > 30,000 (ng/mL)	75.0	72.6	46.9	90	2.7	0.34	7.94 (2.5–25.2)	0.738	.001
CEA < 6.0 (ng/mL) and hyaluronic acid > 30,000 (ng/mL)	68.4	98.3	92.9	90.8	40.2	0.32	127.8 (14.2–1154.3)	0.834	<.001

Pleural HA >30,000 (ng/mL) had a sensitivity of 75.0%, specificity of 72.6%, and odds ratio of 7.94 (95% CI: 2.5–25.2, *P* = .001). When both these parameters were positive, the sensitivity, specificity, positive likelihood ratios, and odds ratios were 68.4%, 98.3%, 40.2, and 127.8 (95% CI: 14.2–1154.3, *P* < .001), respectively (Table 2).

### 3.4 Multiple logistic regression analysis using pleural CEA and HA for discrimination of MM from LC

In the multiple logistic regression analysis, both pleural CEA and HA discriminated MM from LC with hazard ratios of 252.3 (95% CI: 16.4–3888.1, *P* < .001) and 23.6 (95% CI: 2.437–228.1, *P* = .006), respectively (Table 3).

**Table 3:** Multiple logistic regression analysis by using pleural CEA and Hyaluronic acid for discrimination of malignant mesothelioma from lung cancer.

	HR (95% CI)	*P* value
CEA < 6.0 (ng/mL)	252.3 (16.4–3888.1)	<.001
Hyaluronic acid > 30,000 (ng/mL)	23.6 (2.437–228.1)	.006

### 3.5 Comparison of HA levels in pleural effusion of epithelioid and non-epithelioid MM

**Figure 2:**
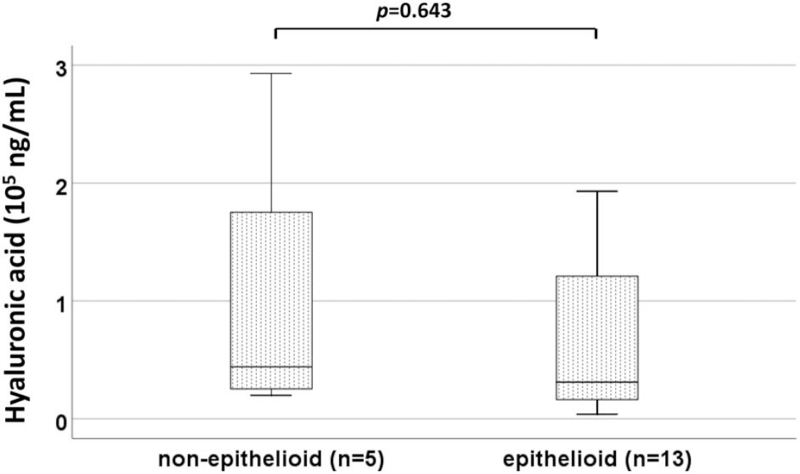
HA levels were comparable between non-epithelioid (n = 5) and epithelioid type (n = 13). HA = hyaluronic acid.

HA was observed in pleural effusion of only 18 patients. HA levels between 5 patients diagnosed with non-epithelioid type (biphasic n = 2, desmoplastic n = 3) (median 44,178 ng/mL, IQR 22,700–23,4138) and 13 patients diagnosed with epithelioid type were comparable (median 31,185, IQR 14,800–15,7000, *P* = .643) (Fig. 2).

## 4 Discussion

This study demonstrated a simple diagnostic method to discriminate MM from LC using pleural CEA and HA obtained from cytologically and pathologically proven cases. The negative result for pleural CEA <6.0 ng/mL has a smaller negative likelihood ratio for MM, and the combination of pleural CEA <6.0 ng/mL and HA >30,000 ng/mL had a high specificity for diagnosing MM.

Welker et al^[6]^ reported that the diagnostic yield (sensitivity and specificity) for HA values of 30,000 ng/mL were 87% and 86%, respectively, and those of 10,000 ng/mL were 39% and 98%, respectively. Fujimoto et al^[7]^ reported that MM should be strongly considered in patients having pleural fluid HA concentrations exceeding 100,000 ng/mL with a sensitivity of 44.0% and specificity of 96.5%. Although the optimal HA threshold depends on the characteristics of enrolled patients (i.e., benign asbestos pleurisy, other malignant diseases, infectious pleuritis, and collagen diseases),^[6–8]^ it would be relatively low against only LC, as in the present study. The concentration of pleural HA in the MM-epithelioid type was higher than that in the sarcomatous type,^[6,7]^ but was comparable in biphasic or other types of MM,^[6]^ as was the case in the present study. Thylen et al^[9]^ demonstrated that elevated concentrations of pleural HA produced by the stromal cells in the surrounding microenvironment, indicated longer survival, which might reflect a functional antagonist against malignant cells that adhere to the mesothelial cells.^[10]^

Although limited biomarkers are available for predicting MM, pleural CEA seems to be a candidate tumor marker.^[2,11]^ Wang et al^[11]^ described that when the cut-off value was set to 1.43 ng/mL, the diagnostic accuracy of pleural CEA for MM among other malignant diseases (i.e., LC, lymphoma/leukemia) had a sensitivity of 83.7%, specificity of 61.1%, positive negative likelihood ratio of 2.15 and 0.27, respectively, similar to the results of this study. Furthermore, the present study revealed that combination methods using pleural CEA and HA can increase the specificity up to 98.3% and a high odds ratio of 127.8.

This study has some limitations. First, it was a retrospective study. Second, a relatively small number of MM patients were enrolled because of its low prevalence. Third, all enrolled patients were cytologically positive (class IV or V), overestimating the diagnostic yield of these pleural markers. Indeed, Radjenovic-Petkovic et al^[12]^ reported the adjunctive effect of pleural CEA on cytological examination, that increased the diagnostic rate for LC from 68% to 85.3%, among patients with benign and malignant pleural effusion.

However, it is noteworthy that positive results of pleural CEA and HA, can be used to diagnose MM. Accumulation of more cases may lead to a simple differential diagnosis of pleural effusion.

## 5 Conclusion

Physicians usually handle malignant pleural effusion, including LC, metastatic pleural diseases, lymphoma/leukemia, and MM. As MM is one of the most challenging malignancies to diagnose, this simple method can have practical applications in both outpatient and inpatient settings.

## Author contributions

**Conceptualization:** Takeshi Saraya, Kosuke Ohkuma.

**Data curation:** Takeshi Saraya.

**Investigation:** Kosuke Ohkuma, Masachika Fujiwara, Haruyuki Ishii.

**Project administration:** Kosuke Ohkuma.

**Writing – original draft:** Takeshi Saraya.

**Writing – review & editing:** Takeshi Saraya.
